# Factors Affecting Usability and Acceptability of an Online Platform Used by Caregivers in Child and Adolescent Mental Health Services: Mixed Methods Study

**DOI:** 10.2196/60042

**Published:** 2024-12-26

**Authors:** Jessica Radley, Jessica Penhallow, Alice Wickersham, Anna Morris, Craig Colling, Johnny Downs

**Affiliations:** 1 Institute of Psychiatry, Psychology and Neuroscience, King's College London, 16 De Crespigny Park, London, SE5 8AF, United Kingdom, 44 7858681876; 2 CAMHS Digital Lab,Institute of Psychiatry, Psychology & Neuroscience, King's College London, London, United Kingdom

**Keywords:** child mental health, caregivers, digital technology, digital health, technology use, digital skill, digital literacy, digital divides, online systems, online survey, pediatric, mental health, usability, platform, survey, questionnaire, children, youth, adolescent, informal care, family care, acceptability, System Usability Scale, SUS, mobile phone

## Abstract

**Background:**

Young people and families endure protracted waits for specialist mental health support in the United Kingdom. Staff shortages and limited resources have led many organizations to develop digital platforms to improve access to support. myHealthE is a digital platform used by families referred to Child and Adolescent Mental Health Services in South London. It was initially designed to improve the collection of routine outcome measures and subsequently the “virtual waiting room” module was added, which includes information about child and adolescent mental health as well as signposting to supportive services. However, little is known about the acceptability or use of digital resources, such as myHealthE, or about sociodemographic inequalities affecting access to these resources.

**Objective:**

This study aimed to assess the usability and acceptability of myHealthE as well as investigating whether any digital divides existed among its userbase in terms of sociodemographic characteristics.

**Methods:**

A survey was sent to all myHealthE users (N=7337) in May 2023. Caregivers were asked about their usage of myHealthE, their levels of comfort with technology and the internet. They completed the System Usability Scale and gave open-ended feedback on their experiences of using myHealthE.

**Results:**

A total of 680 caregivers responded, of whom 45% (n=306) were from a Black, Asian, or a minority ethnic background. Most (n=666, 98%) used a mobile phone to access myHealthE, and many had not accessed the platform’s full functionality, including the new “virtual waiting room” module. Household income was a significant predictor of caregivers’ levels of comfort using technology; caregivers were 13% more likely to be comfortable using technology with each increasing income bracket (adjusted odds ratio 1.13, 95% CI 1.00‐1.29). Themes generated from caregivers’ feedback highlight strengths of digital innovation as well as ideas for improvement, such as making digital platforms more personalized and tailored toward an individual’s needs.

**Conclusions:**

Technology can bring many benefits to health care; however, sole reliance on technology may result in many individuals being excluded. To enhance engagement, clinical services must ensure that digital platforms are mobile friendly, personalized, that users are alerted and directed to their full functionality, and that efforts are made to bridge digital divides. Enhancing dissemination practices and improving accessibility to informative resources on the internet is critical to provide fair access to all using Child and Adolescent Mental Health Services.

## Introduction

### Overview

In the United Kingdom, Child and Adolescent Mental Health Services (CAMHS) offer support for mental health difficulties among individuals under the age of 18 years. In April 2023, around 700,000 children and young people in England accessed mental health services [1]. Young people who are referred to CAMHS have to wait over 12 weeks before receiving treatment [2]. This can be a risky period for young people, with some experiencing deterioration in their mental health while awaiting treatment [3]. It can also lead to fragmented service delivery, since caregivers often contact other children’s mental health agencies while waiting [4]. In addition, the longer a young person spends waiting, the more likely they are to miss their first appointment [5], and the less likely they are to engage in treatment [6], and these missed appointments further increase wait times [7].

As well as reducing waiting times, it is crucial to think about what CAMHS can provide to young people and their parents or carers while they are waiting for assessment or treatment. There is an increasing amount of attention on the potential for digital innovation in mental health services, which can entail delivering therapy and accessing resources via the internet. CAMHS settings are also increasingly considering how digital innovation can support caregivers’ involvement [8]. However, it is essential to consider digital exclusion when discussing and designing digital innovations. Digital exclusion can occur when individuals are not able to access digital services; for example, they may not own a smartphone or computer [9], or lack interest in using technology [9]. More vulnerable groups are also more likely to be affected by digital exclusion, such as those who are older, unemployed, and more socially isolated [10].

Digital innovation can include internet-based portals that inform and engage service users [11]. The myHealthE platform was launched in 2021, as a digital solution to assist collection of caregiver-completed routine outcome measures for young people accessing CAMHS in South London [12]. In England, the National Health Service Outcomes Framework policy [13] recommend the use of routine outcome measures to assess the impact of their interventions in CAMHS [14]. myHealthE facilitates the collection of routine outcome measures from the point of referral, providing clinicians, caregivers and parents a useful way of assessing young people’s current mental health symptoms as well as tracking progress over time [12]. Since myHealthE’s staggered implementation in 2021, over 10,000 caregivers whose young people were referred to South London and Maudsley National Health Service Foundation Trust (SLaM) CAMHS have registered with the myHealthE platform.

In 2022, an extensive public user engagement campaign led by the South London Listens program highlighted the urgent need to provide a better pathway to access information for caregivers and young people while waiting for a CAMHS assessment. A recommendation from the campaign to create a “virtual waiting room” was taken forward by SLaM. After rapid consultation with senior managers, clinicians, and caregivers, a “minimal viable product” online module was developed. This new “virtual waiting room” module hosted new resources and signposting information selected by local CAMHS clinicians, which included, for example, a welcome video explaining the service, psychoeducation videos about low mood and anxiety, as well as external links to UK mental health charities like Mind. This new module was successfully integrated into the myHealthE platform in January 2023 [15]. Enrolled caregivers were then contacted twice (2 weeks apart) in January and February 2023, via personalized email messages to make them aware of the recent updates. Before this update, caregivers reported in a previous small-scale evaluation that myHealthE was generally easy-to-use [12]. No larger scale investigation of its accessibility has since been conducted.

The aim of this study was, first, to conduct a large-scale baseline assessment of the usability and acceptability of the myHealthE platform as well as of its new “virtual waiting room” module. This study also aimed to investigate whether differences in sociodemographic characteristics reveal any digital divides among the caregivers who use myHealthE.

### Research Questions

1. How often, using which devices, and for what purposes are caregivers accessing the myHealthE platform?
2. Are sociodemographic characteristics (ie, age, ethnicity, marital status, and household income) associated with a digital divide, namely, (1) levels of comfort with the internet and technology and (2) perceived usability of the myHealthE platform?
3. How has the myHealthE platform and the inclusion of a virtual waiting room module been received by caregivers?

## Methods

### Ethical Considerations

This project was given approval by SLaM CAMHS Audit and Service Evaluation Committee on April 13, 2023 (Project #236). The project involved a voluntary survey sent to current the current userbase of myHealthE. Participation was anonymous since caregivers did not provide their names on completion and there was no way to link data in order to identify participants. No compensation was provided.

### Participants and Procedures

On May 5, 2023, a text message was sent to all caregivers registered with the myHealthE site containing a link to the online voluntary survey hosted on Qualtrics. The survey was kept open for 6 weeks. There were a total of 29 questions on the survey, displayed on 6 pages. The participants were able to go back and change their answers. The participants saw a bar at the top of the screen indicating their progress through the survey. No incentives for completion were offered. IP addresses were checked to ensure there were no identical addresses.

### Measures

#### Sociodemographic Characteristics

The participants were asked their age range, gender, ethnicity, marital status, and household income.

#### Access of and Attitudes Toward myHealthE

To answer Research Question 1, participants were asked what kinds of digital devices they own and they were asked a single choice question on how frequently they access the myHealthE site, and a multiple choice question on what their reasons were for doing so. They were asked when they first signed up to myHealthE to determine whether they had seen myHealthE before its “virtual waiting room” update in January 2023. For the participants that had signed up to myHealthE before 2023, they were asked if they noticed a change, and then whether they felt the update had improved the myHealthE platform. All participants were asked how helpful and how easy they found it to access the myHealthE resources. The participants were also asked 2 open-ended questions: (1) “Can you give any recent examples of a positive experience of using myHealthE?” and (2) “Do you have any ideas on ways it can be improved?”

The participants were then asked two questions rated on 5-point Likert scales from “very comfortable” to “very uncomfortable”: (1) How comfortable do you feel using technology? and (2) How comfortable are you with accessing the internet? The data for levels of comfort with (1) the internet and (2) technology were polarized rather than normally distributed, and therefore a binary variable was created by grouping the participants who answered “very comfortable” and “slightly comfortable” into one variable and the ones who answered “very uncomfortable” and “slightly uncomfortable” into one variable. Those who answered “neither uncomfortable nor comfortable” were excluded from the analysis.

The System Usability Scale (SUS) was used to measure participants’ views on the usability of the myHealthE platform [16]. It contains 10 items rated on a Likert scale from “Strongly disagree” to “Strongly agree.” It has been shown to have good reliability and face validity [17]. A total of 5 items are phrased in agreement (eg, “I thought the system was easy to use”) and five are phrased in disagreement (eg, “I thought there was too much inconsistency in this system”). The word “system” was replaced with “myHealthE” for this study. Half of the items were reverse-coded so that a higher response indicated a more positive view of the usability of the myHealthE platform. The responses to the items from the SUS questionnaire were converted numerically so the responses to each item ranged from 0 to 4. Then scores were summed and multiplied by 2.5 so that total scores ranged from 0 to 100.

### Analysis

#### Quantitative Data

We conducted statistical analyses to investigate the association between sociodemographic characteristics, levels of comfort with the internet and technology, and perceived usability of myHealthE (Research Question 2).

To examine these digital divides, we conducted a multiple linear regression, with SUS scores as the outcome variable and sociodemographic characteristics of age, gender, ethnicity, marital status, and household income as predictor variables. Gender, ethnicity, and marital status were treated as categorical variables, while age and household income were treated as ordinal variables. Those who were widowed were removed from the marital status variable due to small cell counts (n=3). Similarly, multiple logistic regression models were conducted with levels of comfort with (1) the internet and (2) technology as outcome variables, and the same sociodemographic characteristics as predictor variables. Threshold for statistical significance was set at .05. Complete case analysis was used.

#### Qualitative Data

The qualitative feedback was analyzed using reflexive thematic analysis [18] to answer Research Question 3. Feedback to both open-ended questions was merged and read through twice and then coded to capture the smallest meaningful unit of information. Then codes were grouped conceptually to create initial themes. These initial themes were compared to the original data and redefined and redeveloped until a final set of themes and subthemes were generated and discussed within the research team. This set of themes and subthemes captured how the authors interpreted participants’ feedback to the two questions, with a focus on both participants’ experience with the myHealthE platform as well as digitalization in general.

## Results

The survey was sent out to 7337 users of myHealthE, and a total of 680 individuals responded, giving a 9.27% response rate.

### Demographics

There was a total of 680 participants, of whom 552 (81.2%) completed the survey and 128 (18.8%) provided partial data. The majority of respondents were female, and most were aged in their 30s or 40s. The participants were ethnically diverse: White British was the majority ethnic group (341/644, 53%) followed by Black British or Black African or Caribbean (126/644, 19.6%). The most common marital status was single (274/630, 43.5%; Table 1).

**Table 1.** Sociodemographic characteristics.

Characteristics^a^		Participants, n (%)	
**Gender (n=675)**			
	Women	644 (95.4)	
	Men	31 (4.6)	
**Age (years) (n=664)**			
	25 and younger	17 (2.6)	
	26‐30	34 (5.1)	
	31‐35	107 (16.1)	
	36‐40	125 (18.8)	
	41‐45	168 (25.3)	
	46‐50	119 (17.9)	
	51‐55	70 (10.5)	
	56‐60	24 (3.6)	
**Ethnicity (n=644)**			
	White British	341 (53)	
	Black British or Black African or Caribbean	126 (19.6)	
	Any other White background	77 (12)	
	Mixed or multiple ethnic groups	56 (8.7)	
	Asian or Asian British	27 (4.1)	
	Other ethnic group	17 (2.6)	
**Marital status (n=630)**			
	Single	274 (43.5)	
	Married or civil partnership	254 (40.3)	
	Divorced or dissolved civil partnership	59 (9.4)	
	Separated	40 (6.3)	
	Widowed	3 (0.5)	
**Household income (n=519)** ^b^			
	Below £10,000	128 (24.7)	
	£ 10 ,001-£ 20 ,000	124 (23.9)	
	£ 20 ,001-£ 30 ,000	81 (15.6)	
	£ 30 ,001-£ 40 ,000	48 (9.2)	
	£ 40 ,001-£ 50 ,000	31 (6)	
	£ 50 ,001-£ 60 ,000	31 (6)	
	Above £ 60 ,000	76 (14.6)	

a Data were missing for gender (5/680, 0.7%), age (16/680, 2.4%), ethnicity (36/680, 5.3%), marital status (50/680, 7.4%), and household income (161/680, 23.7%).

b Conversion rate: British £1=US $1.26732.

### myHealthE Access and Usage

The participants were asked what type of internet-enabled devices they owned (Table 2). Over 98% (647/659) owned a smartphone, and 53.4% (352/659) exclusively owned a phone. One percent (8/640) stated they did not own any internet-enabled device. Table 2 describes the frequency of access and reasons for using myHealthE. The majority of participants used myHealthE less than once a month, and a third used myHealthE once a month. Most (528/640, 82.5%) only used myHealthE for one purpose, with the main reason cited as completing outcome measures (575/640, 89.8%).

**Table 2.** myHealthE access and usage.

myHealthE access and usage		Responses, n (%)	
**Type of device (n=659)** ^**a**^			
	iPhone	396 (60.1)	
	Android phone	274 (41.6)	
	Laptop	239 (36.3)	
	iPad or tablet	168 (25.5)	
	PC	65 (9.9)	
	I don’t own any devices	8 (1.2)	
**Number of devices owned (n=651)**			
	1	353 (54.2)	
	2	148 (22.7)	
	3	112 (17.2)	
	4	33 (5.1)	
	5	5 (0.8)	
**Frequency of myHealthE usage (n=640)**			
	Less than once a month	367 (57.3)	
	Once a month	230 (35.9)	
	Once a week	37 (5.8)	
	Multiple times a week	6 (0.9)	
**Number of reasons for using myHealthE (n=640)**			
	1	528 (82.5)	
	2	70 (10.9)	
	3	28 (4.4)	
	4	14 (2.2)	
**Reasons for using myHealthE (n=640)**			
	Completing questionnaires	575 (89.8)	
	Looking at information about CAMHS	109 (17)	
	Looking at resources on myHealthE	54 (8.4)	
	Looking at other organizations	40 (6.3)	
	Other reason	41 (6.4)	

a Participants could select multiple options in response to type of device and reasons for using myHealthE. Data were missing for type of device (21/680, 3.1%), number of devices (29/680, 4.3%), frequency of myHealthE usage (40/680, 5.9%), and reasons for using myHealthE (40/680, 5.9%).

### The myHealthE “Virtual Waiting Room” Module

Very few of the 585 participants who had been registered with the myHealthE site before the update had noticed the “virtual waiting room” update (62/537, 11.5%), and many were not sure whether myHealthE had improved following the update (Table 3). Excluding the “not sure” response option, more participants thought that the update had improved myHealthE than made it worse. Everyone was asked whether they found the new resources helpful and whether they found the resources on the “virtual waiting room” module easy to access. Slightly more participants stated they found the resources more helpful than unhelpful. However, many participants subsequently indicated that had been unaware of the new “virtual waiting room” module resources.

**Table 3.** myHealthE “virtual waiting room” update.

Questions on myHealth update^a^		Responses, n (%)	
**Which year did you first use myHealthE? (n=637)**			
	2021	115 (18.1)	
	2022	205 (32.2)	
	2023	95 (14.9)	
	I don’t know	222 (34.9)	
**Did you notice the update to the myHealthE site? (n=537)**			
	Yes	62 (11.5)	
	Not sure	253 (47.1)	
	No	222 (41.3)	
**Do you feel the new update improved the myHealthE site? (n=277)**			
	Much improved	37 (13.4)	
	Somewhat improved	51 (18.4)	
	Neither improved nor worsened	37 (13.4)	
	Somewhat made worse	2 (0.7)	
	Made much worse	2 (0.7)	
	Not sure	148 (53.4)	
**Do you find the resources on myHealthE helpful? (n=601)**			
	Very helpful	22 (3.7)	
	Somewhat helpful	165 (27.5)	
	Neither helpful nor unhelpful	275 (45.8)	
	Somewhat unhelpful	35 (5.8)	
	Very unhelpful	104 (17.3)	
**Do you find the resources on myHealthE easy to access? (n=601)**			
	Very easy	90 (15)	
	Slightly easy	64 (10.6)	
	Neither easy nor difficult	142 (23.6)	
	Slightly difficult	13 (2.2)	
	Very difficult	6 (1)	
	I did not know about these resources	286 (47.6)	

a All participants (n=680) were presented with the question "Which year did you first use myHealthE?" Only those who answered “2021,” “2022“ or “I don't know” were presented with the question "Did you notice the update to the myHealthE site?" (n=585). Only those who answered “yes“ or “not sure” were presented with the question "Do you feel the new update improved the myHealthE site?" (n=315). Data were missing for ”Which year did you first use myHealthE?” (43/680, 6.3%), ”Did you notice the update to the myHealthE site?” (48/585, 8.2%), ”Do you feel the new update improved the myHealthE site?” (38/315, 12.1%), ”Do you find the resources on myHealthE helpful?” (79/680, 11.6%), and ”Do you find the resources on myHealthE easy to access?” (79/680, 11.6%).

### Digital Divides

#### Levels of Comfort With Technology and the Internet

The participants were asked how comfortable they were with technology and with the internet in general. The majority of participants were slightly (130/659, 19.7%) or very comfortable (261/659, 39.6%) using technology and slightly (88/659, 13.4%) or very comfortable (337/659, 51.1%) using the internet, but around a quarter of participants were slightly (64/659, 9.7%) or very uncomfortable (132/659, 20%) using technology and slightly (39/659, 5.9%) or very uncomfortable (127/659, 19.3%) using the internet.

#### System Usability Scale

For 128 participants, at least one of their responses to the SUS was missing, so they were removed from analyses of SUS. The mean overall SUS score was 62.4 (SD 15.0) with the median score as 60 (IQR 50-72.5), meaning most participants found myHealthE neither easy nor difficult to use, with a slight skew toward participants finding it easy.

#### Examining the Association Between Sociodemographic Factors and Digital Divides

A multiple regression was run with SUS score as the outcome variable and age, gender, ethnicity, marital status, and household income as predictor variables. None of these sociodemographic characteristics were found to be statistically significantly associated with SUS score except those whose marital status was “separated” when compared with those who were “married.” (Table 4). However, a similar difference was not found for those who were “single” or “divorced.”’

Multiple logistic regressions were run with internet comfort and technology comfort as the outcome variables (uncomfortable vs comfortable) and age, gender, ethnicity, marital status, and household income as predictor variables (Table 5). Household income was a statistically significant predictor of how comfortable participants were with the internet and technology, with higher income being associated with higher levels of comfort. When compared with married participants, single and divorced participants were less likely to be comfortable with using technology; however, there was not a significant difference when modeling internet comfort.

**Table 4.** Multiple linear regression analysis between each sociodemographic characteristic (predictor) and System Usability Scale score (outcome), n=461.

Covariate and response category	Estimate (SE)		*P* value
Age	−0.74 (0.54)		.17
**Gender**			
Female	Ref^a^		Ref
Male	−6.76 (4.05)		.10
**Ethnicity**			
White British	Ref		Ref
Black British or Black African or Caribbean	−1.88 (2.07)		.36
Any other White background	1.67 (2.59)		.52
Mixed or multiple ethnic groups	2.51 (2.59)		.33
Asian or Asian British	−0.99 (4.01)		.81
Other ethnic group	−1.79 (4.66)		.70
**Marital status**			
Married	Ref		Ref
Single	−0.48 (1.93)		.80
Divorced	−1.52 (2.91)		.60
Separated	−6.52 (3.07)		.03
Household income	0.36 0.42)		.39

a Ref: reference.

**Table 5.** Multiple logistic regression analysis between sociodemographic characteristics (predictors) with (1) level of comfort with the internet (outcome; “uncomfortable” is the reference category), n=442 and (2) level of comfort with technology (outcome; “uncomfortable” is the reference category), n=444.

Covariate and response category	Levels of comfort with the internet			Levels of comfort with technology		
	Adjusted odds ratio (95% CI)	Standard error	*P* value	Adjusted odds ratio (95% CI)	Standard error	*P* value
Age	1.04 (0.89‐1.21)	0.078	.63	1.05 (0.91‐1.22)	0.075	.52
**Gender**						
Female	Ref^a^	Ref	Ref	Ref	Ref	Ref
Male	1.29 (0.37‐4.43)	0.630	.69	1.53 (0.45‐5.17)	0.623	.50
**Ethnicity**						
White British	Ref	Ref	Ref	Ref	Ref	Ref
Black British or Black African or Caribbean	1.57 (0.89‐2.76)	0.288	.12	1.44 (0.84‐2.48)	0.276	.19
Any other White background	2.17 (0.95‐4.99)	0.423	.07	1.52 (0.72‐3.19)	0.379	.27
Mixed or multiple ethnic groups	1.96 (0.91‐4.21)	0.391	.09	1.73 (0.83‐3.60)	0.374	.14
Asian or Asian British	1.85 (0.50‐6.90)	0.672	.36	3.22 (0.69‐15.10)	0.789	.14
Other ethnic group	0.49 (0.15‐1.58)	0.601	.23	0.34 (0.10‐1.15)	0.617	.08
**Marital Status**						
Married	Ref	Ref	Ref	Ref	Ref	Ref
Single	0.58 (0.34‐1.01)	0.278	.05	0.48 (0.28‐0.82)	0.276	.007
Divorced	0.87 (0.37‐2.04)	0.434	.76	0.43 (0.19‐0.95)	0.410	.04
Separated	1.03 (0.41‐2.57)	0.469	.96	0.47 (0.20‐1.11)	0.438	.09
Household income	1.18 (1.04‐1.34)	0.065	.01	1.13 (1.00‐1.29)	0.063	.04

a Ref: reference.

### Understanding Attitudes Toward myHealthE

There were 142 responses to the question asking about positive experiences and 138 responded to the questions on ideas for improvement. The thematic analysis generated 3 themes and 8 subthemes (Table 6).

**Table 6.** Themes and subthemes.

Themes and subthemes	Illustrative quotations
**Digitalization results in less personalization**	
Digitalization reduces human contact	“A human should talk to me.”“I don’t know if anybody reads the information collected.”
The content isn’t personalized towards me and my child	“Make it personal. You have my details.”“Some questions are about my child’s behavior at school but he doesn’t attend school.”
Barriers to participation in a digital world	“Every time I check for updates to our case I have to reset my password.”“There should [be] good communication through the post!!!”
**A platform with opportunities**	
Room for improvement; introduce more functionality	“Add a medical record scroll down menu containing child’s all reports.”“…a way to communicate or leave a message that can’t be included in a questionnaire.”
Give us more information and support	“Update on local group meetings.”“Tools to assist parents or webinars for accessing tools and services”.
Lack of awareness; missed opportunities	“I wasn’t aware of these new resources mentioned in the questions above. So I recommend promoting those more.”
**Digitalization brings insight and usability**	
Data collation gives information and hope to caregivers	“Completing the questionnaires and seeing the results over time helps me understand my son’s progression.”“It always prompts me and updates me of progress, so I know I haven’t been lost in the system.”
MyHealthE has good usability	“Easy to fill questionnaire.”“Useful links to access support.”

#### Digitalization Results in Less Personalization

This theme captured how among all the benefits of digitalization, caregivers also were left with a lack of personal connection around their child’s journey with health services. This theme contained 3 subthemes.

##### Digitalization Reduces Human Contact

Respondents highlighted how they wanted themselves or their child to have contact with a real person. Caregivers felt that it was their right to be able to speak to someone about their child’s welfare: “A human should talk to me,” and some noted that the format of myHealthE made them feel “disconnected from [their children’s] care.” Caregivers wanted more to come from their completion of questionnaires like having “someone who contacts parents or carers after every test to discuss the results.” Caregivers seemingly struggled to see the direct benefit to their child from their completion of questionnaires, and feared that the information they were providing was not being used: “I don’t know if anybody reads the information collected.” Some caregivers were left with a negative emotional impact on themselves: “I don’t know why I bother filling it in as even though I see how in the red and in desperate need of help my son is, he does not get any help.”

##### The Content Isn’t Personalized Toward Me and My Child

Caregivers seemed to find that myHealthE felt generic and not tailored toward their or their child’s needs. Suggestions of personalization included using caregiver’s and children’s names and other details: “Make it personal. You have my details.” Caregivers felt they could not fully capture the difficulties that their child and their family had been experiencing in just one questionnaire: “Box ticking does not give a full picture of what is happening.” There was also a sense that some questionnaires were not appropriate in terms of age, disability, and school attendance: “my son is a teenager (16) and for me answering questions about sharing toys in school or playing with kids has no sense for me”; “Some questions are about my child’s behavior at school but he doesn’t attend school.”

##### Barriers to Participation in a Digital World

Respondents highlighted their difficulties with accessing the platform: “can never log in… so unsubscribed”; “can’t access the site”; “every time I check for updates to our case I have to reset my password” and with completing the questionnaires: “The forms weren’t fillable on my tech.” Caregivers also struggled to use some of the functionality on the platform: “unable to input my child’s new school.” One respondent also highlighted how digital information is not their preferred method of communication: “there should [be] good communication through the post!!!”

### A Platform With Opportunities

This theme depicted how caregivers identified numerous ways in which myHealthE could be improved and it also seemed that the survey itself had highlighted potential benefits of the platform of which respondents were not previously aware. This theme contained 3 subthemes.

#### Room for Improvement; Introduce More Functionality

Respondents noted that the site had reduced functionality on mobile phones, and one respondent suggested an app: “I struggled to remember where to logon. I [wish] there was an app?” One caregiver also noted it would be valuable to be able to “share to print on android phone.” Users also highlighted the benefits of integration of information from other platforms with myHealthE: “Booking a GP appointment through the app”; “add a medical record scroll down menu containing child’s all reports.”

Some respondents had ideas for how the information from routine outcome measures could be better used: “It would be nice if the information collected [could be] used towards building up reports for the assessment team to have a better understanding to that child.” Caregivers asked for more information about the questionnaires: “state where and who the information from the questionnaires go to.” Respondents also felt that they needed: “a way to communicate or leave a message that can’t be included in a questionnaire.”

#### Give Us More Information and Support

Caregivers wanted more “information on help is available”; one suggestion being information about what is available locally: “update on local group meetings.” Caregivers seemingly wanted to know how they could help their child: “tools to assist parents or webinars for accessing tools and services.”

#### Lack of Awareness; Missed Opportunities

For some respondents, the survey had alerted them to the existence of resources on the myHealthE site: “I wasn’t aware of these new resources mentioned in the questions above. So I recommend promoting those more.” A suggestion was made to “send parents/carers a link or text about it or even create a flyer.”

### Digitalization Brings Insight and Usability

This theme captured how users of myHealthE also noted its benefits. It contained 2 subthemes.

#### Data Collation Gives Information and Hope to Caregivers

Once caregivers on myHealthE complete a questionnaire, they can see their data summarized, as well as seeing any changes from their previous questionnaire. Respondents noted that they like the visualization: “it’s interesting to see the questionnaire results overtime in a graph.” Caregivers noted that they enjoyed how their child’s scores had change: “at the end you see improvement or difficulties. It really picks up on how our home life is at the moment” and that viewing these data also helped them make insight into their child: “completing the questionnaires and seeing the results over time helps me understand my son’s progression.” The questionnaires also seemed to give caregivers hope: “It always prompts me and updates me of progress, so I know I haven’t been lost in the system.”

#### myHealthE Has Good Usability

There were also a number of strengths of the myHealthE platform that were noted by caregivers. Some respondents found it easy to use: “easy to fill questionnaire”; “logging in and answering questionnaires was easy.” Caregivers also highlighted its helpfulness: “it helped me to get more info about how to get help for my child.” Numerous examples of the myHealthE platform were also highlighted as positive: “very helpful and useful information; “useful links to access support”; “simple explanation of resources.”

## Discussion

### Principal Findings

This study surveyed 680 caregivers of young people waiting for treatment in CAMHS to determine the acceptability and use of myHealthE, as well as investigating any digital divides. It found that the majority of caregivers were accessing myHealthE exclusively using a phone, and most accessed the platform less than once a month. Caregivers were not accessing the full functionality of the myHealthE platform, including its new “virtual waiting room” module. Caregivers gave recommendations on how myHealthE could be improved, including ensuring the site is personalized. This study also showed that household income is a significant predictor of digital divides.

### How Often, Which Devices, and for What Purposes Are Caregivers Using myHealthE?

The majority of caregivers indicated that they were only using their phones to access the digital world. Therefore, platforms like myHealthE must ensure they have been designed with mobile use in mind to maximize their accessibility. Any current and future development of digital resources for CAMHS populations must focus on testing new platforms via this medium.

The participants did not use myHealthE particularly frequently, with the majority using it less than once a month. This is not surprising, however, since up until the “virtual waiting room” module was launched, the only use for the system was to complete outcome measures which caregivers were alerted to once every three months. Caregivers were alerted to the new “virtual waiting room” update by text message; however, this survey has demonstrated this was not sufficient, since many were not aware of the full functionality of the platform. This study has, therefore, highlighted the need for developers of digital platforms to establish users’ baseline awareness of a platform’s features. It gives motivation for continual improvement and an increase in dissemination of features on platforms like myHealthE to promote awareness of and engagement with digital resources.

### Does a Digital Divide Exist Between the Caregivers Who Access myHealthE?

We did not find strong evidence for associations between sociodemographic characteristics and System Usability Scale scores. However, we found household income to be a predictor of comfort with the internet and technology, with higher income predicting greater levels of comfort. Household income is an indicator of social deprivation and vulnerability, a known source of digital divide [10]. It is important to note that we still found this to be a factor, even within a sample of individuals whose level of comfort with the internet and technology was sufficiently high to own a device and complete an online survey. There was also some evidence that individuals who were single or divorced were less comfortable with technology compared to married individuals.

It is important to note that this study investigated digital divides using an online survey, meaning it is possible that other sociodemographic characteristics would have been found to predict usability and comfort if a nondigital survey was used. For example, despite ethnicity not being found to be a significant predictor of responses to the SUS or levels of comfort with the internet or technology, it is a known factor in literature concerned with digital divides, and must continue to be addressed in the development of digital platforms [19]. In addition, other variables not measured by this study, such as education level, have been found to influence digital divides [20]. Future studies should continue to investigate digital divides using study designs with a range of data collection methods, and should consider investigating the interaction between sociodemographic variables such as household income and ethnicity.

### How Has myHealthE and Its New “Virtual Waiting Room” Module Been Received by Caregivers?

Respondents’ views on digitalization were polarized. Its pitfalls were made clear and there was a general sense of depersonalization. Caregivers felt that the platform was generic and not tailored toward their family. Positive aspects included the visualization of change in their child’s score on outcome measures. Suggestions from caregivers included using their names and their young people’s names in notifications. Customizability has also been named as a recommendation by participants in other studies of digital platforms [21]. Digital platforms in mental health settings could also improve their user experience by tailoring questionnaires and content to suit certain mental health presentations, neurodiversity, and different age ranges of children.

The System Usability Scale measured participants’ views on the usability of myHealthE. The average score was 62.4, which represents “high marginal” acceptability [22] and is lower than the previous evaluation of myHealthE using SUS, which found that a sample of 8 participants rated it at 78 [12]. However, this study had a much larger sample size, and it is likely therefore to be more representative of the true response of the myHealthE userbase. It is essential that digital platforms use measures like the SUS to evaluate their usability in a standardized manner; however, this is not currently common practice [23].

Caregivers were asked whether they thought myHealthE had been improved by the “virtual waiting room” update in January 2023, which expanded the platform from exclusively collecting routine outcome measures, to also including information about CAMHS, mental health resources, and information on other organizations. Only 11.5% (62/537) of respondents indicated they had noticed the update to the site. This demonstrates that the two alerts sent to caregivers were not sufficient to notify them of this update. Therefore, despite efforts being made to build and populate the new “virtual waiting room’ module, insufficient effort had been made to promote awareness of and engagement in these resources. Therefore, digital platforms should consider a variety of methods to notify their userbase of updates, such as using both text and email as well as suggesting users visit new webpages when they are on the site.

### Strengths and Limitations

This study is the first evaluation of the myHealthE platform in its current form using both quantitative and qualitative analyses, and has resulted in specific recommendations for the improvement of all digital platforms used by CAMHS populations.

A major limitation of this study is that, by using an online survey, our ability to measure digital divides was limited, and likely affected by sampling and response bias. The appraisal of comfort with the internet and technology are particularly likely to be overestimated, since by the study’s design, participants were all able to respond to an online survey using an internet-enabled device. In addition, fewer than 10% of myHealthE’s total userbase responded to the survey, indicating that our results are likely representative of those who are more motivated to complete online surveys. Another limitation was the lack of male respondents. This limitation stems from the underlying electronic health record system in SLaM, which permits only one contact detail for a primary caregiver to be listed, and female caregivers are often preferentially recorded in these fields. Therefore, findings relating to gender should be interpreted with caution.

### Conclusions

This study provides a baseline assessment of the acceptability and usability of the myHealthE platform. It provides useful guidance for all health care providers for children and young people on developing online resources for caregivers and young people waiting to access mental health services. It also considered barriers to accessing these platforms. We found that although there was, in general, good acceptability and usability of myHealthE, there were still numerous recommendations made by users and opportunities for improvements. For example, this study highlighted the importance of ensuring digital platforms are mobile friendly. It also highlighted potential gaps between development and dissemination of new information to service users; it is not simply enough to create new materials, we must ensure that service users are fully informed. Finally, it is essential that platforms like myHealthE are not solely designed for individuals with high digital literacy, but also consider how individuals might be being excluded through digitalization. Bridging digital divides, particularly those observed among different sociodemographic groups, such as different income levels, is crucial.

## Abbreviations

CAMHS: Child and Adolescent Mental Health Services
NIHR: National Institute for Health and Care Research
SLaM: South London and Maudsley National Health Service Foundation Trust
SUS: System Usability Scale
